# Esmolol inhibits cognitive impairment and neuronal inflammation in mice with sepsis-induced brain injury

**DOI:** 10.1515/tnsci-2022-0297

**Published:** 2023-07-25

**Authors:** Yanpeng Li, Junli Ma, Jianjun Diao, Wei Chen, Zhihua Wang

**Affiliations:** Department of Emergency, Shanghai Pudong Hospital, Fudan University Pudong Medical Center, 2800 Gongwei Road, Pudong, Shanghai 201399, China; Department of Cardiovascular Medicine, Shanghai Pudong Hospital, Fudan University Pudong Medical Center, 2800 Gongwei Road, Pudong, Shanghai 201399, China

**Keywords:** sepsis, esmolol, cognitive impairment, neuronal inflammation, lipopolysaccharide

## Abstract

Sepsis is a potentially fatal organ failure resulting from a dysregulated host response to infection. It can be a substantial financial burden on families and society due to the high cost of medical care. The study aims to investigate the protective roles of Esmolol in mice with sepsis-induced brain injuries against cognitive dysfunction and neuronal inflammation. Male C57BL/6J mice were intraperitoneally injected with LPS (10 mg/kg, L2630, Sigma) to establish a septic encephalopathy model. Esmolol (15 mg/kg/h, HY-B1392, MedChemExpress) was subcutaneously infused using osmotic mini-pumps for 6 h before LPS injection. Morris water maze and novel object recognition tests evaluated LPS-induced cognitive impairment and behavioral phenotypes. Cytokines and protein expression were assessed using ELISA assay and RT-qPCR. Esmolol treatment potentially improved cognitive impairment in septic mice. Esmolol administration markedly diminished the abnormal hippocampal neuronal structure, and the expression of interleukin (IL)-1β, IL-6, and tumor necrosis factor-α was significantly downregulated in the hippocampal tissue. Esmolol treatment significantly reduced apoptotic TUNEL-positive cells and reversed the related gene expression (BAX and BCL-2). The effects of esmolol on the reactive oxidative species and oxidative stress markedly reduce malondialdehyde MDA content and increase superoxide dismutase and catalase in hippocampal tissues. In addition, esmolol significantly reduced the percentage and density of Iba-1 + microglia in septic mice. Our results demonstrated that esmolol potentially improved cognitive impairment and neuronal inflammation in mice with sepsis-induced brain injury.

## 1 Introduction

Sepsis is a systemic inflammatory condition associated with the emergence of several organ disorders, including brain dysfunction [1]. Clinically, sepsis increases the blood–brain barrier’s permeability, decreases cerebral blood flow, and promotes the flow of neurotoxic agents and neuroinflammation [2]. Sepsis is a severe public health problem affecting people worldwide and is linked to death, morbidity, and cognitive impairment [3]. However, the patient’s quality of life is severely influenced by cognitive impairment. In addition, there are limitations and challenges in correctly assessing the cognitive and sensory functions of sepsis patients [4]. However, the mechanisms of cognitive impairment and neuroinflammation in sepsis patients remain unclear.

The specific mechanism of sepsis still needs to be fully discovered, despite the increased study attention given to the pathophysiology of the condition. An excessive inflammatory response was induced in response to invasive infections, which exacerbated sepsis [5]. Pathogen-associated chemicals, such as endotoxin, stimulated innate immune cells and swiftly recruited and activated neutrophils and macrophages to remove bacteria and heal damaged tissue [6]. Interleukin (IL) and tumor necrosis factor-α (TNF-α) are pro-inflammatory cytokines that are released in large quantities during sepsis and further contribute to numerous cell death and organ malfunction [7]. Additionally, sepsis patients frequently have an unbalanced level of oxidants and antioxidants, which promotes cell death and tissue damage in vulnerable brain areas, such as the hippocampus [8]. Therefore, the balanced levels of the antioxidant superoxide dismutase (SOD) and lipid peroxidation malondialdehyde (MDA) are effective indicators in determining and measuring the degree of oxidative damage. The pathophysiology of sepsis is mainly dependent on mitochondrial-mediated apoptotic pathways, in addition to oxidative stress [9]. Therefore, it was essential to generate effective therapies against oxidative stress and anti-apoptosis in the pathogenesis of sepsis to stop the cognitive impairment caused by sepsis.

Neuroinflammation is accompanied by injury, infection, toxicity, and autoimmunity of the central nervous system [10]. Inflammatory reactions cause the production of cytokines and growth factors that favorably influence the tissue after damage when transiently stimulated. Moreover, chronic or uncontrolled inflammatory reactions can negatively affect the pathological development of neurodegenerative diseases, including cognitive impairment, Parkinson’s disease, multiple sclerosis, and amyotrophic lateral sclerosis [11,12]. Of the neurodegenerative diseases, cognitive impairment is remarkable. Several types of cognitive impairment include dementia, mild cognitive impairment, subjective cognitive impairment, and normal cognitive decline with aging [13]. The most prevalent form of dementia, Alzheimer’s disease, is accompanied by significant cognitive impairment [14]. The prevalence of cognitive impairment is currently increasing on a global scale. However, there is no therapy that works effectively. Thus, highly effective therapeutic medications need to be investigated immediately.

A most prevalent therapy for cardiovascular disorders, β-receptor blockers, enhance myocardial remodeling, metabolism, and immunological regulation while lowering heart rate and serving as an antiarrhythmic [15]. Esmolol is a highly selective β1 receptor blocker with a short half-life that may be delivered through the drip. Its advantages include rapid onset, high tolerance, and straightforward management, making it the most used preparation in critical care medicine [16]. New investigations demonstrate that esmolol may alter the perioperative pain response [17] and minimize anesthetic needs [18], while not being previously recognized to have anesthetic or analgesic effects [19]. However, the effect of esmolol on cognitive impairment and neuroinflammation remains unknown. Therefore, this study aims to investigate and provide precise information about the protective effects of esmolol regarding cognitive impairment and neuroinflammation with sepsis-induced brain injury and to explore the specific molecular mechanism.

## 2 Materials and methods

### 2.1 Animals and treatment

**Figure 1:**
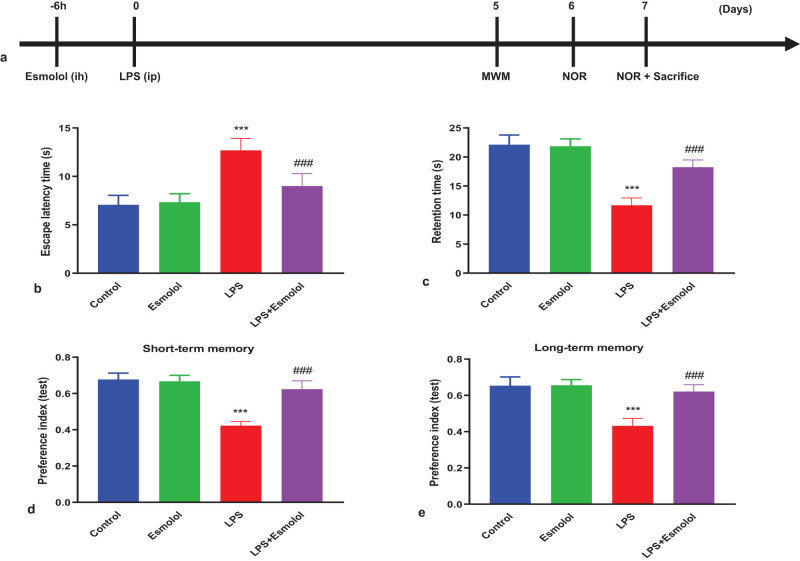
Esmolol ameliorates cognitive deficits in septic mice. Mice were randomized and divided into control, esmolol, LPS, and LPS + esmolol groups. Mice were subcutaneously infused with saline or esmolol (15 mg/kg/h) for 6 h and then were intraperitoneally injected with saline or LPS (10 mg/kg). (a) Timeline of interventional procedure and cognitive tests after esmolol infusion and LPS injection. The MWM test was carried out 5 days after LPS injection to determine (b) escape latency and (c) retention time. The NOR test was trained on day 6 after LPS injection and tested at 30 min or 24 h later. The time spent with a novel object (preference index) was measured to evaluate (d) short-term memory and (e) long-term memory. Data were shown as mean ± SD (*n* = 8 per group) and were analyzed by one-way ANOVA. ***P < 0.001 vs control group; ###*P* < 0.001 vs LPS group.

Male C57BL/6J mice (6–10 weeks old, 20–25 g) were used to establish the sepsis model. All mice were maintained in a controlled environment free of pathogens with a 12 h light/dark cycle. Mice were intraperitoneally injected with LPS (10 mg/kg, L2630, Sigma) to establish a septic encephalopathy model. Esmolol (15 mg/kg/h, HY-B1392, MedChemExpress) was subcutaneously infused using osmotic mini-pumps for 6 h before LPS injection. Mice in control and LPS groups received the same saline volume. The cognitive and behavior tests were performed 5, 6, and 7 days after the LPS injection, and then mice were anaesthetized with 1% pentobarbital sodium and killed to obtain hippocampal tissues (Figure 1a).

### 2.2 Morris water maze (MWM) test

The MWM test was used to evaluate mice’s learning and memory abilities. Before the experiment, mice were trained to assess their motor function for continuous 4 days (days 1–4). In each trial, mice were given 60 s to find a hidden platform in a white pool (120 cm in diameter) placed 1 cm below the water’s surface. Then, the platform was removed and the mice were left to find the platform for 60 s. The time for mice to find and climb onto the immersed platform (escape latency) and the time spent in the target quadrant (retention time) were recorded.

### 2.3 Novel object recognition (NOR) test

On day 6 after the LPS injection, each mouse was placed in a square space (50 cm × 50 cm × 50 cm) for familiarization and discrimination. In the familiarization phase, each mouse spent 10 min in the field with two identical objects (A1 and A2). After 30 min and 24 h, each mouse was returned to the open field where one of the familiar objects (A2) was replaced by a novel object (A3). In the discrimination phase, each mouse explored objects for 10 min, and the time of exploring each object was recorded. Preference indexes were calculated as the time spent on a novel object (A3) normalized to the total time spent on A1 and A3. Tests measured at 30 min and 24 h after the familiarization phase were used to evaluate short- and long-term memory, respectively.

### 2.4 Biochemical analysis

The hippocampal tissue was homogenized (10%, w/v) in normal cold saline, and the lysate was collected for enzyme activity analysis. According to the manufacturer’s instructions, the levels of MDA and SOD were determined using the nitroblue tetrazolium assay using an MDA assay kit (S0131S, Beyotime, Shanghai, China) and a SOD assay kit (S0109, Beyotime, Shanghai, China).

### 2.5 Enzyme-linked immunosorbent assay (ELISA)

Interleukin-1β (IL-1β; MLB00C; R&D Systems), interleukin-6 (IL-6; M6000B; R&D Systems), and tumor necrosis factor-α (TNF-α; MTA00B; R&D Systems) levels were determined following the manufacturer’s instructions. Serum was collected from mice on day 7, and the hippocampal tissue was homogenized (10%, w/v) in normal cold saline to obtain the supernatant. A microplate reader was used to determine the plates’ absorbance. The corresponding standard curve evaluated the concentrations of IL-1β, IL-6, and TNF-α.

### 2.6 Histology

To examine the apoptosis of hippocampus tissues, Nissl staining was used. Nissl solution (C0117, Beyotime, Shanghai, China) was used to stain the hippocampus tissue section for 5 min at 37°C. The sample was dried after being cleaned with 95% ethyl alcohol. The tissue sample was sealed with neutral balsam after being cleaned twice with xylene. Under an optical microscope (Olympus-BX51), a tissue section was examined. The average number of surviving neurons in the hippocampus CA1 was counted at a magnification of 200×, and the amount of neuronal density loss was computed.

### 2.7 Immunohistochemistry

The hippocampal tissue was embedded in paraffin and cut into 5 μm serial slices after being treated with 4% paraformaldehyde. The tissues were deparaffinized in xylene, placed on positively charged slides, and rehydrated. Hematoxylin–eosin, Masson’s trichrome, or rabbit polyclonal antibodies against IL-1β (1:500; sc-52012, Mouse monoclonal, Santa Cruz) were used to stain the slides. After 20 min incubation at 37°C, the secondary antibody was biotinylated horseradish peroxidase. The slides were counter-stained after being cleaned. Images were captured with a 200 X zoom lens from Nikon, Tokyo, Japan, and were then examined using ImageJ (NIH, Bethesda, MD, USA).

### 2.8 Immunofluorescence

Frozen sections of the hippocampal tissue from mice were obtained. TUNEL (C1086, Beyotime), dihydroethidium (DHE) (S0063, Beyotime), and Iba-1 antibody (1:200, sc-32725, mouse monoclonal, Santa Cruz) were applied after washing with PBS three times. The cells were then counterstained with DAPI and examined under an inverted microscope (IX51, Olympus, Japan). TUNEL and DHE were performed according to the manufacturer’s instructions.

### 2.9 Statistical analysis

The results were replicated at least three times and reported as mean ± standard deviation (SD). SPSS 20.0 (SPSS, Chicago, IL, USA) was used for the statistical analyses. One-way ANOVA was used to analyze the differences between three or more groups. Statistical significance was set as *P* < 0.05.


**Ethical approval:** The research related to animal use complied with all the relevant national regulations and institutional policies for the care and use of animals. This study was performed ethically in accordance with the World Medical Association Declaration of Helsinki (CPP 2012/19 2012-A00190-43).

## 3 Results

### 3.1 Esmolol ameliorated cognitive deficits in septic mice

The MWM test was performed to clarify the effects of esmolol on spatial learning and memory impairments in septic mice. The latency to reach the hidden platform (escape latency) and the time spent in the target quadrant (retention time) were measured. The escape latency of LPS mice was significantly longer than that in control mice. Esmolol administration significantly decreased the escape latency compared with LPS mice (Figure 1b). The total time spent in the target quadrant was considerably reduced in LPS mice compared to control mice. Esmolol significantly increased the time duration compared with LPS mice (Figure 1c). The NOR test was carried out to evaluate the mice’s working memory and exploratory behaviors. LPS mice exhibited a significantly decreased preference index in short- and long-term memory compared to control mice. In contrast, esmolol remarkably mitigated sepsis-induced recognition memory impairments compared to LPS mice (Figure 1d and e).

### 3.2 Esmolol attenuated LPS-induced hippocampal injury and inflammation in septic mice

**Figure 2:**
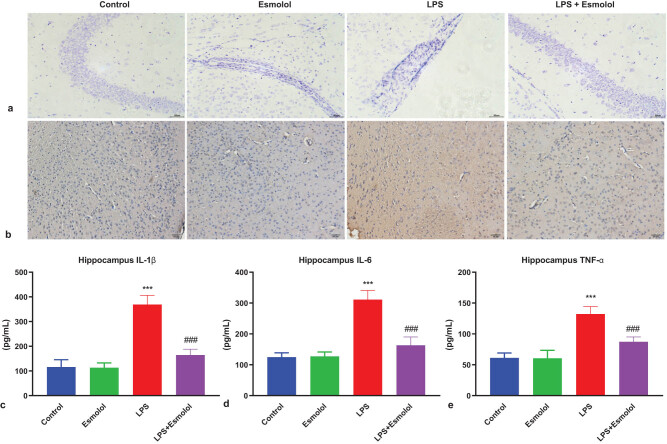
Esmolol ameliorates injury and inflammation in the hippocampus of septic mice. (a) Representative images of Nissl staining in hippocampal tissue following LPS injection (magnification, 200×; scale bar: 50 μm). (b) Representative image of IL-1β expression in the hippocampal CA1 region of LPS mice by immunohistochemistry (magnification, 200×; scale bar: 50 μm). RT-qPCR was used to determine the mRNA expression levels of (c) IL-1β, (d) IL-6, and (e) TNF-α in the hippocampal tissues of mice. Data were shown as mean ± SD (*n* = 8 per group). ***P < 0.001 vs control group; ###*P* < 0.001 vs LPS group.

To investigate the morphologic changes in the hippocampal tissue, we performed Nissl staining 7 days after LPS injection. The neuronal structure of the hippocampal CA1 area in control mice was complete and clear, with orderly arranged cells. In LPS mice, the hippocampal neuronal structure was unclear and disordered. Esmolol administration markedly diminished these abnormal changes (Figure 2a). We then performed immunohistochemistry to determine the inflammatory response in the hippocampal tissue. LPS injection significantly increased the protein expression of IL-1β, which was attenuated by esmolol (Figure 2b). Moreover, RT-qPCR was performed to determine the mRNA expression of genes associated with pro-inflammatory cytokines. LPS administration significantly increased the mRNA expression of IL-1β, IL-6, and TNF-α in hippocampal tissue, and was significantly downregulated by esmolol (Figure 2c–e).

### 3.3 Esmolol attenuated LPS-induced hippocampal apoptosis in septic mice

**Figure 3:**
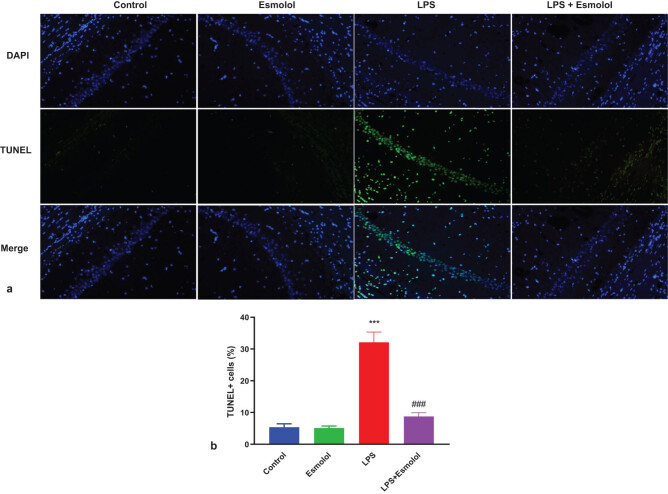
Esmolol inhibits LPS-induced apoptosis in the hippocampus of septic mice. (a) Representative image of TUNEL staining in hippocampal tissue (Magnification, 200×; Scale bar: 50 μm). (b) The TUNEL + cells were quantified in hippocampal tissue and normalized to DAPI + cells. ***P < 0.001 vs control group; ###*P* < 0.001 vs LPS group.

The hippocampal tissue of mice was stained with TUNEL to assess the apoptosis extent. The green fluorescence intensity in the hippocampal tissue was more vigorous in LPS mice compared to control mice, and this green fluorescence was attenuated by esmolol (Figure 3a). Quantification analysis confirmed the suppressive effect of esmolol on apoptosis in the hippocampal tissue, as evidenced by reduced TUNEL + in LPS + esmolol mice compared to LPS mice (Figure 3b).

### 3.4 Esmolol suppressed oxidative stress in the hippocampus of septic mice

**Figure 4:**
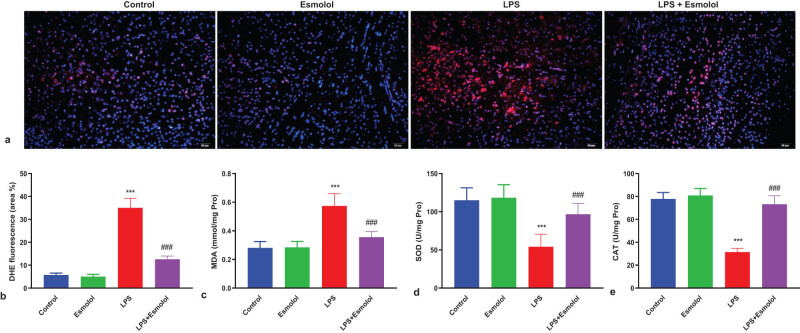
Esmolol suppresses oxidative stress in the hippocampus of septic mice. (a) Representative images of hippocampal tissue stained with DHE (magnification, 200×; scale bar: 50 μm). (b) The extent of cellular ROS was quantified by calculating the percentage of DHE fluorescence area. The levels of (c) MDA, (d) SOD, and (e) CAT were measured in the hippocampal lysate. Data were shown as the mean ± SD (*n* = 8 per group). ***P < 0.001 vs control group; ###*P* < 0.001 vs LPS group.

The intracellular reactive oxidative species (ROS) was measured by staining the hippocampus with DHE. LPS significantly increased the hippocampal DHE fluorescence intensity and area and were markedly reversed by esmolol administration (Figure 4a and b). Then, the three key biomarkers of oxidative stress were measured in the lysate of the hippocampus. LPS increased the MDA content and was further attenuated by esmolol (Figure 4c). In contrast, SOD and catalase (CAT) activities were reduced by LPS and further improved by esmolol (Figure 4d and e).

### 3.5 Esmolol inhibited microglia activation in septic mice

**Figure 5:**
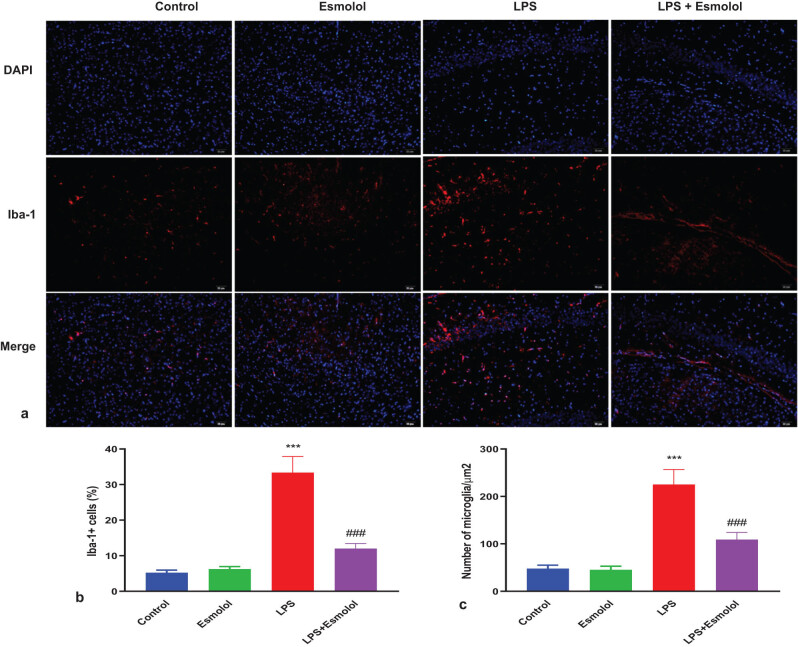
Esmolol reduces microglia activation in septic mice. (a) Representative Iba-1 immunofluorescence staining in the hippocampus of mice treated with esmolol beginning at 6 h before LPS injection (magnification, 200×; scale bar: 50 μm). Statistical analysis of (b) Iba-1 + cell and (c) density of microglia in the hippocampus. Data were shown as mean ± SD (*n* = 8 per group). ***P < 0.001 vs control group; ###*P* < 0.001 vs LPS group.

To explore changes in microglia activation in the hippocampus of mice, we stained microglia with Iba-1 and DAPI by immunofluorescence. LPS enhanced the red fluorescence of Iba-1 in the hippocampus, markedly attenuated by esmolol (Figure 5a). Quantification analysis showed that the percentage and density of Iba-1 + microglia were significantly increased in the LPS mice compared with the control mice. Esmolol significantly reduced the percentage and density of Iba-1 + microglia (Figure 5b and c).

## 4 Discussion

The present study investigated the potential protective effect of esmolol on cognitive impairment and neuroinflammation in mice. Our study showed that esmolol significantly improved cognitive impairment, reduced LPS-induced hippocampal injury and inflammation, inhibited LPS-induced hippocampal apoptosis, suppressed oxidative stress, and attenuated microglia activation in septic mice. Therefore, esmolol could have protective impacts against LPS-induced cognitive impairment and neuronal inflammation in mice.

Sepsis is a severe public health problem worldwide and is associated with mortality, morbidity, and cognitive impairment [3]. Cognitive impairment results from neuronal damage, which also plays a critical role in sepsis-induced neuronal injury, that impairs cognitive functions [20]. The cognitive function resembles clinical settings and is frequently assessed using the Morris water maze and neurological function [21]. However, our results showed that esmolol therapy potentially reduced the escape latency compared with LPS mice (Figure 1b) and increased the time duration compared with LPS mice (Figure 1c). In contrast, esmolol significantly reduced sepsis-related deficits in recognition memory compared to LPS mice (Figure 1d and e). Thus, esmolol treatment potentially improved cognitive deficits in septic mice.

Brain edema is accompanied by IL-1β, IL-6, and TNF-α cytokines, exacerbating mitochondrial dysfunction and increasing ROS levels, leading to neuronal inflammation [22]. Different inflammatory diseases have been linked to the production of ROS; cells produce ROS as a kind of host defense response [23]. Tyrosine phosphatases and other cellular signaling proteins are oxidized by ROS, which worsens endothelial dysfunction [24]. Reactive nitrogen species are produced when ROS and NO interact, dismutating superoxide by SOD [25]. Additionally, ROS and SOD act as inflammatory mediators and stimulate neuronal damage and inflammation. However, the current study demonstrated that esmolol significantly improved LPS-induced hippocampal injury (Figure 2a) and potentially inhibited the expression of IL-1β, IL-6, and TNF-α in the hippocampal tissue (Figure 2c–e). Thus, esmolol reduced LPS-induced hippocampal injury and inflammation in septic mice.

BAX, BCL-2, and caspase-3 mediated neuronal apoptosis [26]. Imbalanced levels of anti- and proapoptotic proteins facilitate apoptosis. The anti-apoptotic protein Bcl-2 inhibits mitochondrial-dependent apoptosis by decreasing its activation [27]. The caspase pathway activation by elevated caspase-3 activity promotes neuronal apoptosis. Neuronal apoptosis is stimulated by sepsis-induced neuronal damage and is prevented by decreased apoptosis [28]. In this study, we observed that esmolol potentially reduced LPS-induced hippocampal apoptosis in septic mice (Figure 3a and b).

The hippocampus was stained with DHE to quantify the intracellular ROS. The data of the present study showed that esmolol treatment considerably reduced the hippocampus DHE fluorescence intensity and area, which were dramatically elevated by LPS (Figure 4a and b). The lysate of the hippocampus was then tested for three key oxidative stress biomarkers. Esmolol further reduced the MDA content, while LPS increased the MDA content (Figure 4c). In contrast, LPS increased SOD and CAT activities, which were then further enhanced by esmolol (Figure 4d and e). Thus, esmolol significantly attenuated the oxidative stress in the hippocampus of septic mice.

Neuronal inflammation and microglial activation are significant pathogenic aspects of brain injury. After cranial irradiation, which produces pro-inflammatory cytokines and damages immunity homeostasis, neurogenesis, vascular integrity, and cognitive function, microglia operate as early responders since they are the innate immune cells in the brain [29,30,31]. In addition, neuropathic pain is a result of activated microglia [32]. However, our study used immunofluorescence to stain microglia with Iba-1 and DAPI to examine changes in microglia activation in the mice hippocampus. Esmolol significantly decreased the red fluorescence of Iba-1 in the hippocampus, which was augmented by LPS (Figure 5a). According to the quantification analysis, the proportion and density of Iba-1 + microglia were considerably higher in the LPS animals than in the control mice. Esmolol dramatically decreased the proportion and density of Iba-1 + microglia (Figure 5b and c). Thus, esmolol potentially inhibited microglia activation in septic mice.

There are several limitations to our study. (a) We did not assess the dose-dependent effect of esmolol on the septic mice model. Future research should explore different esmolol dosages to see whether the esmolol’s inhibitory effect on cognitive impairment and neuronal inflammation was dose-dependent. (b) In clinical research, the mechanisms of esmolol are complicated and ambiguous. However, the main goals of this study were to assess how esmolol affected cognitive impairment and neuronal inflammation in mice. (c) Even though esmolol can attenuate cognitive impairment and improve neuronal inflammation in mice, inhibiting them might not be possible if they already exist. However, esmolol might be a critical preventative and therapeutic approach for neuronal inflammation and cognitive impairment in pre-clinical and clinical settings, although further research is required to validate this.

In conclusion, esmolol significantly alleviated cognitive impairment and neuronal inflammation in mice with sepsis-induced brain injury. Further study is needed to confirm whether esmolol could be an effective preventive therapeutic strategy for cognitive impairment and neuronal inflammation in pre-clinical and clinical settings.
